# RETRACTED: Godugu et al. Nanoformulated Ajwa (Phoenix Dactylifera) Bioactive Compounds Improve the Safety of Doxorubicin Without Compromising Its Anticancer Efficacy in Breast Cancer. *Molecules* 2020, *25*, 2597

**DOI:** 10.3390/molecules30091996

**Published:** 2025-04-30

**Authors:** Kavitha Godugu, Ali H. El-Far, Soad Al Jaouni, Shaker A. Mousa

**Affiliations:** 1 Pharmaceutical Research Institute, Albany College of Pharmacy and Health Sciences, Rensselaer, NY 12144, USA; Kavitha.Godugu@acphs.edu; 2 Department of Biochemistry, Faculty of Veterinary Medicine, Damanhour University, Damanhour 22511, Egypt; ali.elfar@damanhour.edu.eg; 3 Department of Hematology/Pediatric Oncology, Yousef Abdulatif Jameel Scientific Chair of Prophetic Medicine Application, Faculty of Medicine, King Abdulaziz University, Jeddah 21589, Saudi Arabia; saljaouni@kau.edu.sa

The Journal retracts the article “Nanoformulated Ajwa (Phoenix Dactylifera) Bioactive Compounds Improve the Safety of Doxorubicin without Compromising Its Anticancer Efficacy in Breast Cancer” [1] cited above.

Following the publication of a correction [2] to this article [1], further concerns were brought to the attention of the Editorial Office by Albany College of Pharmacy and Health Sciences relating to the validity of sub-panels presented in Figure 6.

Adhering to our standard procedure, an investigation was conducted by the Editorial Office and Editorial Board and supported by information provided by the Albany College of Pharmacy and Health, the Editorial Board agreed with the institutional assessment that the replacement images provided in the above-mentioned correction could not be appropriately validated due to the lack of supporting raw material and thus could not be considered as appropriately resolving the identified concern. As a result, the Editorial Board has lost confidence in the reliability of the findings and decided to retract this publication [1]. This retraction supersedes the previously published correction [2] and is carried out as per MDPI’s retraction policy (https://www.mdpi.com/ethics#_bookmark30).

This retraction was approved by the Editor-in-Chief of the journal *Molecules*.

The authors did not provide a comment on this decision.
